# A Method for Non-Rigid Face Alignment via Combining Local and Holistic Matching

**DOI:** 10.1371/journal.pone.0159376

**Published:** 2016-08-05

**Authors:** Yang Yang, Shaoyi Du, Zhuo Chen

**Affiliations:** The School of Electronic and Information Engineering, Xi’an Jiaotong University, Xi’an, ShaanXi, China; Chinese Academy of Sciences, CHINA

## Abstract

We propose a method for non-rigid face alignment which only needs a single template, such as using a person’s smile face to match his surprise face. First, in order to be robust to outliers caused by complex geometric deformations, a new local feature matching method called K Patch Pairs (K-PP) is proposed. Specifically, inspired by the state-of-art similarity measure used in template matching, K-PP is to find the mutual K nearest neighbors between two images. A weight matrix is then presented to balance the similarity and the number of local matching. Second, we proposed a modified Lucas-Kanade algorithm combined with local matching constraint to solve the non-rigid face alignment, so that a holistic face representation and local features can be jointly modeled in the object function. Both the flexible ability of local matching and the robust ability of holistic fitting are included in our method. Furthermore, we show that the optimization problem can be efficiently solved by the inverse compositional algorithm. Comparison results with conventional methods demonstrate our superiority in terms of both accuracy and robustness.

## 1 Introduction

Face alignment plays an important role in many applications, such as face recognition [1, 2], performance analysis [3] and object tracking. It is an interesting problem and has been paid more attentions in computer vision. The objective of face alignment is to find a transformation between two images, so that all image pixels can be matched to the same semantic locations. In recent years, face alignment has gained significant progress in both theory and practice. However, the problem of only using a single template in face alignment still remains unexplored. The reason could be that the facial appearances exhibit quite differently even if two images are from a same person but with different expressions. In addition, we do not have any prior knowledge about the specific expressions.

The basic face model is one of the most important part in this study. In literature, the well known Active Appearance Model (AAM) [4] solves the problem by synthesizing both an appearance model and a shape model for target face. Many methods [5–8] have been proposed based on the AAM to improve the alignment accuracy and robustness. However, when an unseen target face has a large appearance variation from the training set, this kind of generative appearance model has poor performance [9]. Some discriminative face alignment methods [10–12] using the regression model directly maps the appearance of an image to the landmarks. They can be successful in face alignment with desired accuracy and robustness as long as there are enough training data. However, they are also critical by the requirement of a large number of training data for regression.

Given only a single template as the appearance information, the current face alignment methods can be roughly classified into two categories: holistic fitting method and local matching method. In the first category, Lucas-Kanade algorithm (LK) [13] is the most popular technique. The matching error under the transformation parameter is directly minimized in the object function. LK can reach sub-pixel accuracy. But the parameter optimization process is based on the gradient descent method which requires good initialization for getting global optimal parameters. Yang et al. [14] proposed a face alignment method using a single template as an extension of the LK. A facial deformation model added as the regularization term is built based on the difference between neutral and expression faces in the linear space. Later Yang et al. [15] also presented a non-linear facial deformation model and a deformation guider in the image alignment formulation. In these methods, the shape parameter is formulated into a more compact space which improve the matching accuracy. But these two methods only effect when the template face is with neutral expression. Zhu et al. [16] proposed a unsupervised face alignment method by nonrigid mapping. A face region is represented by mesh and the mapping between two face images is parameterized by the vertex coordinates. This method is also based on the LK and robust to appearance variations, but it aligns a holistic face area other than each landmark of facial components.

On the other hand, there is a growing focus on local matching methods [17] [18]. This kind of methods use local features to build correspondences between template and target images. The registration process is simplified as calculating the transformation parameters with known correspondence. There are many feature extraction methods such as Scale-Invariant Feature Transform (SIFT) [19], HoG features [20] and Local Binary Features [21]. Wang et al. [22] proposed a patch-based exhaustive search method that could align faces with a single template. Local matching method is more flexible to overcome the limitation of bad initialization. However, since they ignored the global image appearance, the fitting results are dependent too much on the local similarity. Especially, for non-rigid image alignment, the deformation has many degrees of freedom. A large number of local salient features should be found which contain outliers. But, the traditional similarity measure such as Sum of Squared Differences or Normalized Cross-Correlation measure is not robust to outliers caused by nonrigid deformation. A novel similarity measure called Best-Buddies Similarity (BBS) [23] has been proposed recently for its good performance of object detection in the wild. In BBS, the Best-Buddies Pairs (BBP) between two images are found. However, when BBP is used for face images, the matching number decreases under complex geometric deformations, which will influence the precision of non-rigid registration.

In this paper, we propose a method for non-rigid face alignment which only needs a single template, such as using a person’s smile face to match his surprise face. Local matching features are introduced into the process of holistic fitting. Both the flexible ability of local matching and the robust ability of holistic fitting are included in our method. We use the basic patch-based local presentation. To avoid negative influence caused by local mismatching, a new local matching search method called K Patch Pairs (K-PP) is proposed. Inspired by the BBP, K-PP is to find the mutual K nearest neighbors between two images. It is robust to outliers caused by complex geometric deformations. Our optimization process is efficient under the Inverse Compositional algorithm. Furthermore, the global rigid transformation is also considered in the alignment framework. Experimental results compared with conventional methods demonstrate the effectiveness of the proposed approach.

The remainder of this paper is organized as follows. In Section 2, we present the problem formulation by a face shape model as the background. In Section 3, we provide the proposed local K Patch-Pair search method. Next, the details about building the local regularization term, parameter optimization and implementation details are given. The method is tested by comparison experiments in Section 4. Finally, conclusions are given in the last section.

## 2 Face Alignment Problem with a Shape Model

Given a template face image *T* and a target face image *I*, a common way is to formulate the alignment problem in the framework of Lucas-Kanade (LK) algorithm [13]. The goal is to minimize the sum of squared error between two images by finding a warping function **W**(**x**; **p**) with the parameter **p** for all pixel coordinates **x** in the fitting area. The formulation form of the LK algorithm is

$$E_{c}=\sum_{x}{∥T\left(x\right)-I\left(W\left(x;p\right)\right)∥}^{2}.$$ (1)

Let $s^{p}=\left\{s_{1}^{p},s_{2}^{p},...,s_{N}^{p}\right\}$ be the shape feature vector of the *p*th face example. *N* is the number of annotated face landmarks and **s**_*i*_ = [*x*_*i*_, *y*_*i*_] recording the geometric location of the *i*th landmark. The Point Distribution Model [24] (PDM) is applied on the feature matrix composed by all examples to acquire their mean vector $\bar{s}$ and the basis matrix of variation **Q**.

After the model training on face examples, any shape feature **s** can be approximated by the corresponding low-dimensional parameter **p** as:

$$s=\bar{s}+Qp.$$ (2)

With the geometric locations of face landmarks, face region can be separated by computing Delaunay triangulations. In that case, **W**(**x**;**p**) is often chosen as the piecewise affine warp in Eq (1) as the non-rigid transformation.

By optimizing the Eq (1), the face shape can be reconstructed by Eq (2). However, this kind of holistic fitting method is very sensitive to the initial parameters, which is not robust to the non-rigid facial deformation. We are going to add the local matching knowledge to guide the fitting method in the following sections.

## 3 Proposed Method

In this section, We firstly explain our way to search local matching features. Then the optimization algorithm is proposed followed by a description of our formulation model with local constraints. Finally, the novel algorithm and its implementation details are summarized.

### 3.1 Local K Patch Pairs (K-PP) Search

For facial image, the appearance features around some landmarks change a lot during the nonrigid deformation. Local feature matching may have many outliers. Recently, a kind of similarity measure method, called Best-Buddies Pairs (BBP), has made a great process for overcoming the influence of outliers. However, in order to get a high precision of image alignment, two aspects should be considered, 1) the local mismatch rate should be low, and 2) the number of corresponding points should be high. They are two contradictory aspects. Though the BBP can be applied to the object detection successfully, it only ensures the first aspect. This implies that the BBP is not very suit for non-rigid image alignment. To deal with this problem, we propose a K Patch Pairs (K-PP) search method to balance the above aspects by weights.

Taking one landmark as example, finding it’s corresponding location by K-PP contains three steps:

1. Choose patch and search region
  In the template image, we set a *n* × *n* patch around this landmark. The feature of this landmark *Pa* is represented by their RGB values or gray values of all pixels in the patch. We define the search region as neighbor regions *m* × *m* (*m* ≥ 2*n*) of this landmark. Similarly, in the target image, taking the current or initial landmark as the central, the search region is its *m* × *m* neighbor regions. We break the search regions both in the template and target images into *n* × *n* patches by sliding window. Let *Tpset* = {*Tp*_1_, *Tp*_2_, …, *Tp*_*n*_*p*__} be the patch set in the template search region, and *Ipset* = {*Ip*_1_, *Ip*_2_, …, *Ip*_*n*_*p*__} be the patch set in the target search region. *n*_*p*_ is the patch number in search regions.
2. Find corresponding location
  The conventional BBP method find mutual nearest neighbors (NN) for feature points. In K-PP, we improve the method by finding the mutual *k*NN for landmarks patch.
  Firstly, the *k* nearest neighbors (*k*NN) {*Ip*_*j*_, *j* = 1, …, *K*} is found in *Ipset* for *Pa*. Then, for each *Ip*_*j*_, if its *k*NN in *Tpset* includes *Pa*, *Ip*_*j*_ and *Pa* are defined as the mutual *k*NN.
  Then we find the patch *Ip*_*j*_ with minimum number *k* by Eq (3).
  $$\hat{k}=\arg\min_{k}\left\{kNN\left(Tp,Ipset\right)=Ip_{j}\wedge kNN\left(Ip_{j},Tpset\right)=Tp\right\}.$$ (3)
  This patch’s central is defined as the corresponding location of the landmark.
3. Get weight
  To evaluate the similarity of K-PP, a weight *w* is defined based on the parameter $\hat{k}$. This weight parameter is useful for the alignment formulation to distinguish the importance of each local corresponding landmarks.
  $$w=(K-\hat{k})/K.$$ (4)

**Link to the BBP**: Our K-PP is a natural extension of the BBP. But our method is different as follows: (1) The BBP searches the corresponding location for every patch in the template. The K-PP focus on one patch around each landmark. (2) The BBP only considers the mutual NN, which is a particular case of K-PP when *K* = 1. (3) The K-PP also give a definition of weight for balancing the similarity and the matching number.

**Fig 1:**
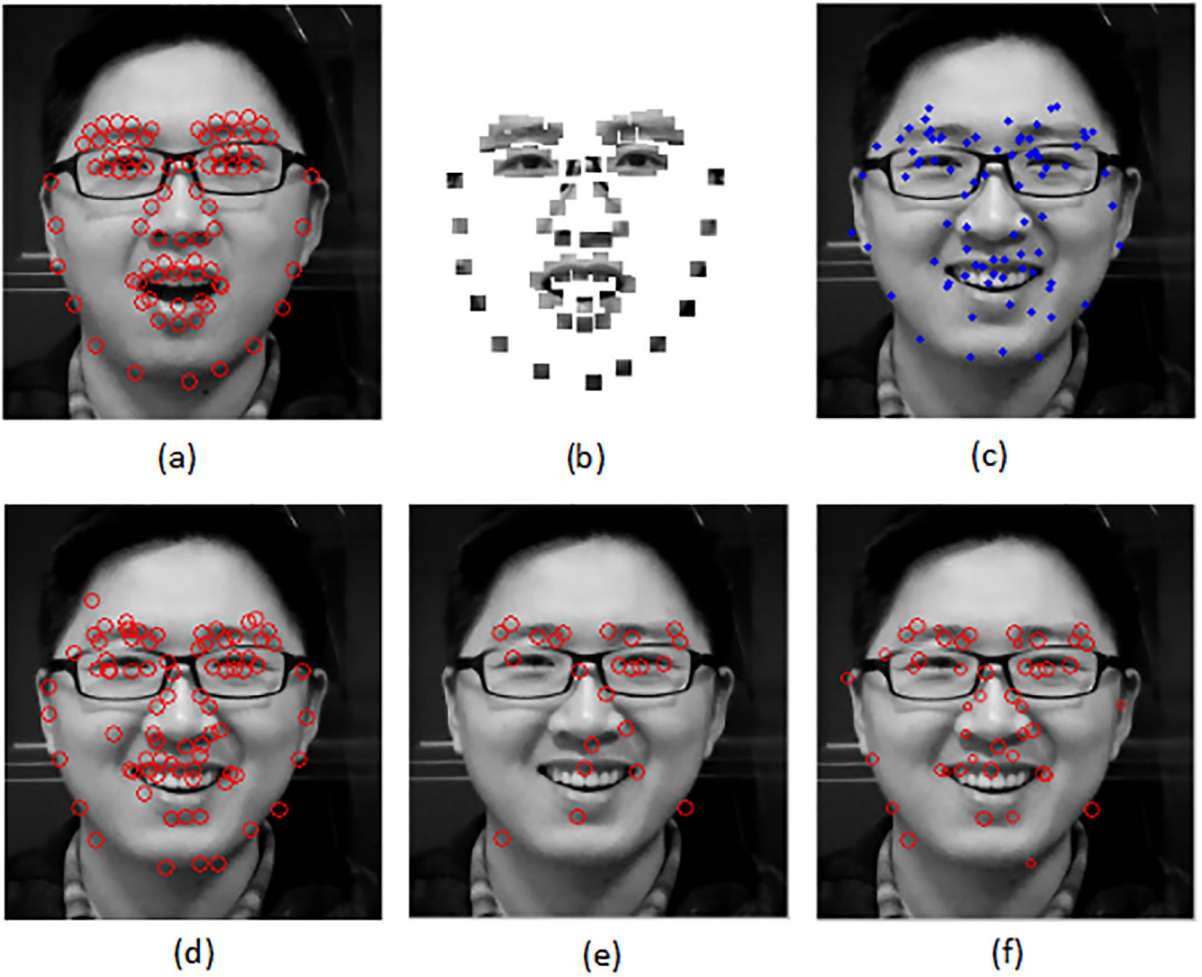
Local matching results. (a) The template image and landmarks. (b) The patch representation of (a). A 9 × 9 patch is set around each landmark. (c) The target image with initial landmark. (d) The local matching results by NN. (e) The local matching results by 1-PP (KPP, K = 1). (f) The local matching results by 5-PP (KPP, K = 5). The size of the circle presents the weight value.

Fig 1 shows the results of local matching search by three different methods. The 9 * 9 patch representation is given in Fig 1(b) around the landmarks. A set of initial landmarks which are far away from the real positions are localized on the target face in Fig 1(c). The local search results are compared in Fig 1(d)–1(f). We can see that the number of corresponding points are the most by the NN method, since it finds the nearest point for every point without other constraints. But there are many mismatching results which will give negative influence to image alignment. The 1-PP which is also the BBP method has a strict rule for choosing matching point to ensure the correct matching. So only a very small number of the landmarks can find the corresponding points. Our K-PP result can get more matching, since we extend the mutual NN in BBP to mutual *k*NN. They are not only with high correct rate, but also have the weights to reflect the matching similarity.

### 3.2 Matching Model and Optimization Algorithm

The traditional LK method shown as Eq (1) is easily fallen into a local minimum solution, thus we modified the method by adding a regularization term about the local matching constraint as the prior knowledge. With the local search result by K-PP, we construct the related regularization term. The main purpose here is to minimize the distance from shape features to local search results with weights.

$$E_{L}=\sum_{i}w_{i}{\left(s_{i}-s_{Li}\right)}^{2},$$ (5)

where **s**_*Li*_ is the feature vector of the *i*th landmark of by K-PP search. If we cannot find the K-PP for some feature point, the corresponding vector **s**_*Li*_ is kept as its initial value, but the weight *w*_*i*_ is set to be zero.

In order to be presented and calculated easily, let $R\left(p\right)=\bar{s}+Qp-s_{L}$. Then *E*_*L*_ can be formulated by matrix.

$$E_{L}=R{\left(p\right)}^{T}KR\left(p\right)$$ (6)

where **K** ∈ *R*^2*N* × 2*N*^ is the weight matrix according to *w*_*i*_ defined as a diagonal matrix.

Combined with Eq (1), the proposed formulation method for face alignment with local constraints is

$$E\left(p\right)=E_{c}\left(p\right)+\lambda E_{L}\left(p\right),$$ (7)

where λ balances between two terms.

Considering the computational efficiency, we adopt the idea of Inverse Compositional(IC) algorithm [13] to minimize *E*(**p**). The optimization process is to update the optimal increments △**p**, and then to update warping function **W**(**x**;**p**). By switching the role of template and target, the Hessian matrix could be pre-computed and re-used. Note that here the inverse incremental parameters is related to the regulation term.

$$\begin{array}{c} E\left(\Delta p\right)=\sum_{x}{∥T\left(W\left(x;\Delta p\right)\right)-I\left(W\left(x;p\right)\right)∥}^{2}\\ +\lambda R{\left(p+\frac{\partial p^{\prime }}{\partial \Delta p}\Delta p\right)}^{T}KR\left(p+\frac{\partial p^{\prime }}{\partial \Delta p}\Delta p\right), \end{array}$$ (8)

where **p**′ denotes the updated warping parameters, such as

$$W\left(x;p^{\prime }\right)=W\left(W{\left(x;\Delta p\right)}^{-1};p\right).$$ (9)

The function *E*(△**p**) is nonlinear with respect to △**p**, which can be linearized by taking the first order Taylor expansion.

$$\begin{array}{ccc} E(\Delta p) & \approx & \sum_{x}{∥T+\nabla T\frac{\partial W}{\partial p}\Delta p-I\left(W\left(x;p\right)\right)∥}^{2}\\ & + & \lambda { [R\left(p\right)+\frac{\partial R}{\partial p}\frac{\partial p^{\prime }}{\partial \Delta p}\Delta p]}^{T}K [R\left(p\right)+\frac{\partial R}{\partial p}\frac{\partial p^{\prime }}{\partial \Delta p}\Delta p]. \end{array}$$ (10)

According to definition of *R*(**p**), we have $\frac{\partial R}{\partial p}=Q$. We set **s**′ as the reconstructed shape feature from **p**′ according to Eq (2). Combining Eq (9), it is computed as **s**′ = **W**(**s** − **Q**△**p**;**p**). Applying the chain rule, we have

$$\frac{\partial p^{\prime }}{\partial \Delta p}=\frac{\partial p^{\prime }}{\partial s^{\prime }}\frac{\partial s^{\prime }}{\partial \Delta p}=-Q^{-1}\frac{\partial W(x;p)}{\partial x}Q.$$ (11)

Let $B=\frac{\partial R}{\partial p}\frac{\partial p^{\prime }}{\partial \Delta p}=-\frac{\partial W(x;p)}{\partial x}Q$. The Hessian matrix is

$$H=\sum_{x}\nabla T{\frac{\partial W}{\partial p}}^{T}\nabla T\frac{\partial W}{\partial p}+\lambda B^{T}KB.$$ (12)

Finally, the solution of the above incremental parameter can be stated as:

$$\Delta p=H^{-1}\left(\sum_{x}(I\left(W\left(x;p\right)-T\right)-\lambda_{c}B^{T}KR\left(p\right)\right).$$ (13)

### 3.3 Implementation

We design the proposed face alignment algorithm as an iteration process with local search and parameter optimization. We also notice that the PDM for shape features is trained on the normalized face examples. There is no rigid transformation factor in model parameter **p**. To improve the model’s adaptation to rotation and translation transformation, the local matching points by K-PP are applied again. Given two sets of corresponding landmarks *M* = {**m**_1_,**m**_2_, …,**m**_*N*_*m*__} and *D* = {**d**_1_,**d**_2_, …,**d**_*N*_*m*__}, the rigid transformation parameters have been solved by the classic Iterative Closet Point algorithm. Thus we only give the conclusion here. The rotation matrix **R**_*θ*_ is calculated by a construction matrix **C** and its singular value decomposition (SVD) [25].

$$C=\frac{1}{N_{m}}\sum_{i=1}^{N_{m}}m_{i}d_{i}^{T},$$ (14)

C=UΛV. (15)

$$R_{\theta }=VU^{T}.$$ (16)

And the translation matrix **Tr** is computed by

$$Tr=\frac{1}{N_{m}}\left(\sum_{i=1}^{N_{m}}m_{i}-\sum_{i=1}^{N_{m}}R_{\theta }d_{i}\right).$$ (17)

The rigid transformation parameters are estimated at the beginning of each iteration process. The proposed algorithm is summarized as follows:

1. Feature Patch Generation: Given a template image, we obtain a set of feature patches around their landmarks.
2. K-PP Search: The proposed local K-PP search is performed for each feature patch.
3. Rigid Normalization: The rigid transformation parameters are computed by Eqs (16) and (17). The current landmarks are normalized by rigid transformation.
4. Local Constraint Construction: The weights *w*_*i*_ is calculated according to Eq (4). The regularization term about local matching constraint is constructed by Eq (6).
5. Parameter Optimization: The shape parameter **p** is optimized by Eq (13). The features of current landmarks are updated.

Steps 2–5 are repeated until converged.

Note that after each iteration process, the local search regions are also changed with the current landmarks. In that case, the K-PP results and the regularization term are updated correspondingly.

## 4 Experimental Results

In the experiments, we select 263 face images from 80 subjects in the Cohn-Kanade Facial Expression Database [26] and 20 face images from 10 subjects captured by ourself. Their expressions include surprise, smile, sad, angry and neutral expression. Big facial deformation has more challenge, such as using a closed mouth to matching a big open mouth. So the faces we selected from the database are with the biggest extent of each expression of every person. All faces are annotated 80 landmarks manually. The training images are regularized by the positions of eye corners to 256 × 256 pixels. To accommodate global lighting variation, the illumination intensity of face region is normalized in advance. In our experiments, the shape model remains 90% principle components. The regularization coefficient λ is set to 10.

We conduct two sets of experiments: the performance of local matching search and final alignment results. The target image has different expressions with the template image. Since the appearance changes, so the two image are mismatch. In the first set of experiments, our goal is to find an optimal parameter *K* in the K-PP method to balance the similarity and the number of local matching. So the matching accuracy and matching number of different methods are exhibited to show the performance. In the second set of experiments, we compare our method with three different methods which could be used for non-rigid face alignment only using a single template. They are the Lucas-Kanade method (LK), the LK method combined with the nearest neighbor search (LK+NN), the LK method combined with the BBP (LK+BBP) and ours: LK with K-PP search (LK+K-PP).

### 4.1 Performance of Local Matching Search

In order to evaluate the performance of local matching search, we compare the K-PP method with different parameters: *k* = 1 (1-PP) which is also the BBP method, *k* = 2 (2-PP), *k* = 4 (4-PP), *k* = 6 (6-PP), and the nearest neighbor search method (NN). 20 template and target image pairs are adopted in local matching experiments. Each image pair is the same person with different expressions. Training examples are not needed here. To test the robustness of each method, we randomly initialize the locations of landmarks by rotation and translation deformation and add noise in two ways: (a) rotated 5 degrees and randomly perturbed by Gaussian noise *N*(15, 10), (b) rotated landmarks with 10 degrees and then perturbed landmarks by Gaussian noise *N*(10, 10).

To quantitatively evaluate the accuracy of the algorithm, we calculate the Root Mean Square Error (RMSE) of testing results with manually annotated ground truth landmarks in Eq (18).

$$RMSE=\frac{\sum_{i=1}^{N}Dist\left(Result_{i}-Groundtruth_{i}\right)}{2*N},$$ (18)

where *N* is the number of landmarks and *Dist*(·) is the Euclidean distance.

**Fig 2:**
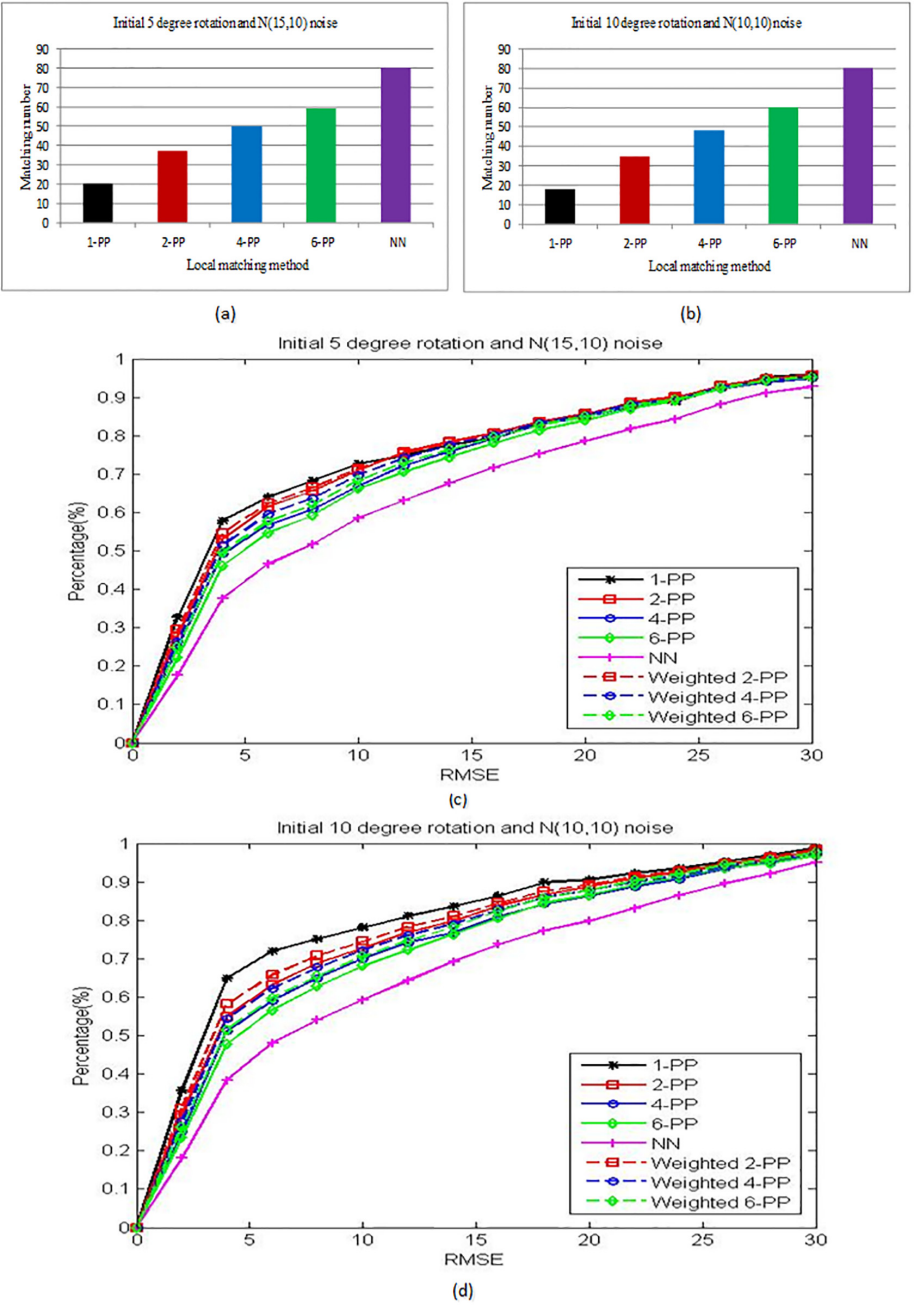
Local matching search results of different methods including the nearest neighbor (NN) method, 1-PP (BBP), 2-PP, 4-PP and 6-PP method. (a) and (b) show the average matching numbers when the initial location of landmarks are rotated 5 degrees and randomly perturbed by Gaussian noise *N*(15, 10), and rotated 10 degrees and randomly perturbed by Gaussian noise *N*(10, 10), respectively. (c) and (d) are the cumulative RMSE curves with corresponding initial locations of (a) and (b). The x-axis denotes the RMSE threshold and y-axis denotes the percentage of test images which are less than each threshold. The solid curves are the K-PP results without weight or the weights are equal to 1. The dashed curves are the K-PP results with weights.

The number of local matching points is another criterion to evaluate the performance. As shown in Fig 2(a) and 2(b), we can see that the matching number increases when the parameter *k* becomes bigger. The 1-PP method finds the smallest number of matching points. The NN method can find the matching point for every landmark. So its matching number equals to the annotated landmark’s number. The percentages of accumulative RMSE curves are drawn in Fig 2(c) and 2(d). We can see that the matching accuracy decrease when the *K* becomes bigger. The results by 1-PP method has the highest matching accuracy. And the results by NN method has the lowest matching accuracy. We also evaluate the effectiveness of weight matrix defined in Eq (4). The comparing results are shown as the solid curves and dashed curves respectively in Fig 2(c) and 2(d), which demonstrate that the weighted matching results have higher accuracy.

From the above experiment, the results verify that the matching number and the matching accuracy are two contradictory aspects in face alignment. But for the face alignment problem, both of them affect the final matching results. So in the following experiments, we use the weight matrix to balance them and choose *k* = 4 as a trade-off parameter in the K-PP.

### 4.2 Face Alignment Result

**Fig 3:**
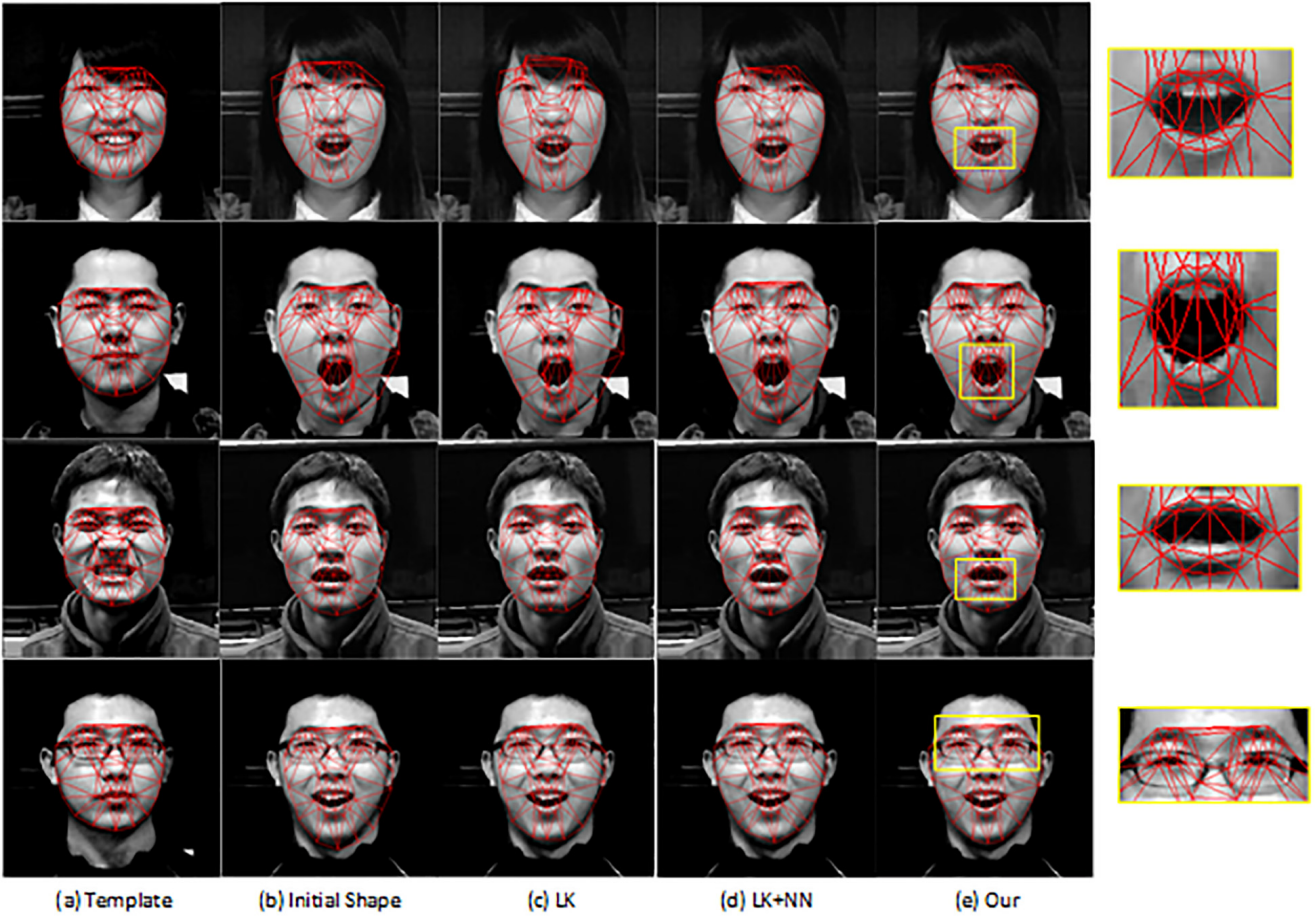
Comparison of the face alignment using LK, LK+NN and our methods on real images. (a) Template with annotated landmarks. (b) Target image with initial shape parameter. (c) The results of Lucas-Kanade method. (d) The results of LK method with the nearest neighbor search. (e) The results of our method, which is Lucas-Kanade method with KPP search (*K* = 4).

To demonstrate the generative of the proposed method, the appearance of testing images captured by ourself are Asian, which are totally different from the training database [26]. Fig 3 shows some examples of face alignment results. We compare our method with LK method and LK+NN method. The templates and target images with initial locations are shown in Fig 3(a) and 3(b). The template and target faces have very different expressions. So the facial appearances change a lot caused by geometric deformations. The results by different methods are shown in Fig 3(c)–3(e). We can see that the LK method is based on the gradient descent algorithm, which is easy to be influenced by local texture leading to a local minimum solution. Better results can be obtained by combining with the NN search which consider both the holistic appearance and local features. But a few mismatch results by the NN search give negative influence to alignment results. For example, for the third person, some local patches of the mouth are mismatched to the nose. So the upper lip is dragged to the mouth by the LK+NN method. But our proposed method performs best which is robust to these outliers.

To quantitatively evaluate the accuracy of the algorithm, we calculate the Root Mean Square Error(RMSE) of testing results with manually annotated ground-truth. All 263 images in database [26] are tested. A leave-one-out cross-validation scheme is adopted throughout the quantitative experiments. When one image is used as target face, a corresponding image from the same person but with different expressions is selected as the target image. We compare four different methods: the proposed LK+4-PP method(Our), LK method, LK+BBP method and LK+NN method. Three kinds of noise are added to the positions of landmarks for initialization: (a) perturbed landmarks by Gaussian noise *N*(0, 10), (b) rotated *N*(0, 5) degrees and randomly perturbed by Gaussian noise *N*(15, 10), (c) rotated *N*(0, 10) degrees and randomly perturbed by Gaussian noise *N*(10, 10). Since the optimal parameter is the shape model parameter *p* in Eq (2), the initial landmarks are first projected to the low-dimensional linear space. So the actual initial position is normalized by the shape model.

**Fig 4:**
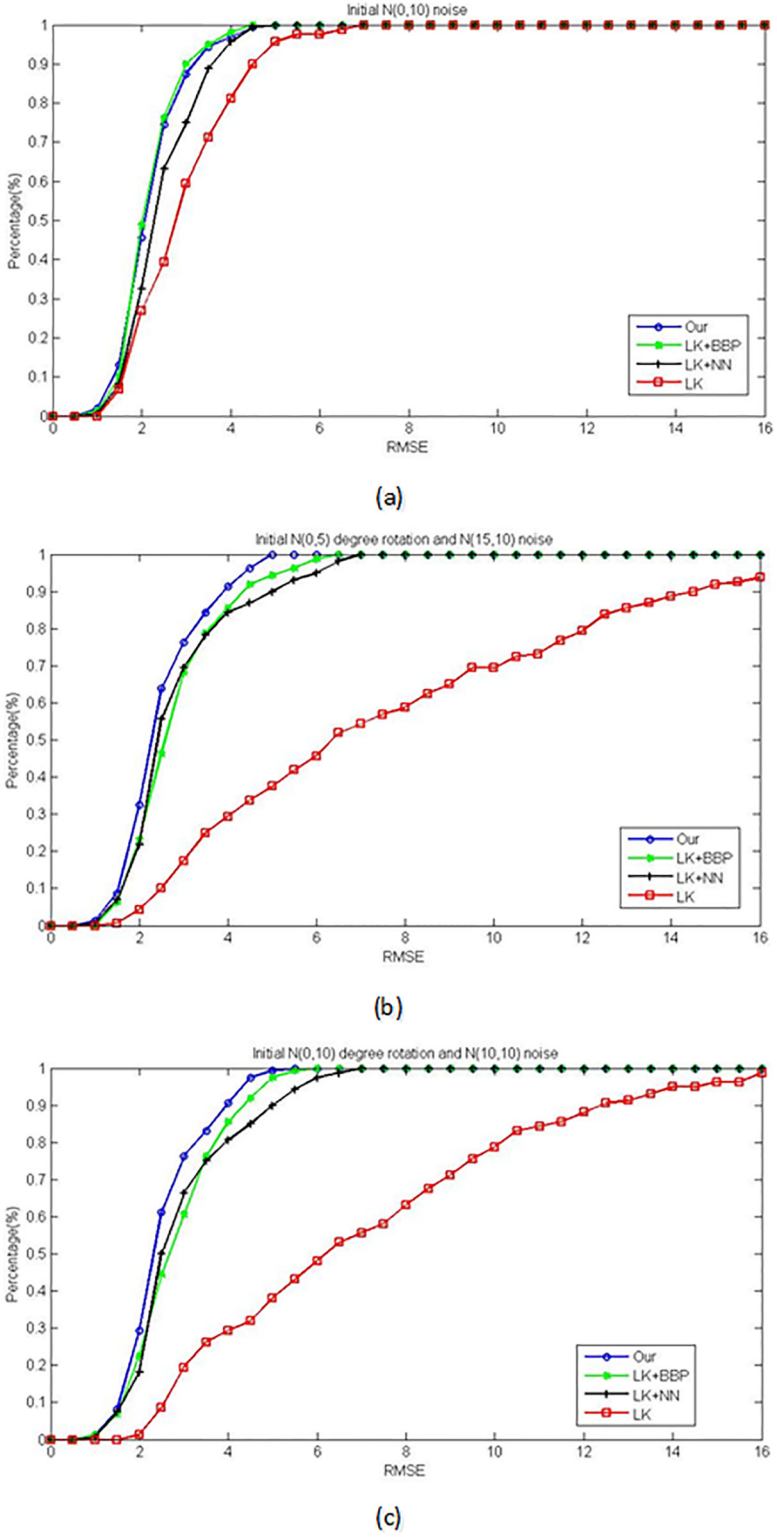
The cumulative RMSE curves with different initial parameters by four methods: Ours (LK+4-PP), LK+BBP, LK+NN and LK. (a) The results with initial Gaussian noise *N*(0, 10). (b) The results with initial rotated *N*(0, 5) degrees and randomly perturbed by Gaussian noise *N*(15, 10). (c) The results with initial rotated *N*(0, 10) degrees and randomly perturbed by Gaussian noise *N*(10, 10).

The accumulative RMSE curves are shown in Fig 4. For the small initial noise in Fig 4(a), our method and LK+BBP method have better results. The LK+BBP method is a little better than ours. We analyse the reason is that, when the noise is small, the LK+BBP method is similar like the LK method with very good initial value. Then the optimization process can find the global optimal parameters. When given big noise, the comparing results are shown in Fig 4(b) and 4(c). Since there is no rigid transform parameter in the LK method, it plays very bad performance. But with the local search method in the LK+NN and our methods, the global rigid fitting could be applied naturally. Our method is not only robust to outliers but also have enough number of local matching prior, so our method is robust to the initial noise and has the best performance among the comparing methods.

Different facial components have different extents of deformations when doing expressions. We have noticed that the alignment results on facial local regions have much differences caused by various appearance changes. We record the average error in terms of the RMSE for eyes, eyebrows, nose, mouth and face contour in Table 1. The lower face region including mouth and contour may have bigger matching errors than upper face region. We can see our method still get the best results for each facial component. The average matching errors are lower than 7 pixels which outperforms those competing methods.

**Table 1:** The RMSE results for each facial component by different methods.

	*N*(0, 5) rotation and *N*(15, 10) noise				*N*(0, 10) rotation and *N*(10, 10) noise			
	LK	LK+NN	LK+BBP	Our	LK	LK+NN	LK+BBP	Our
Eyes	5.68	3.65	3.46	**3.37**	5.30	3.77	3.53	**3.45**
Eyebrows	17.18	4.99	5.55	**4.79**	19.07	4.69	5.24	**4.58**
Nose	10.14	4.54	4.73	**4.16**	10.11	4.16	4.50	**3.88**
Mouth	16.83	8.29	6.69	**5.80**	19.51	8.37	6.83	**6.13**
Contour	17.36	7.40	7.36	**6.89**	19.46	6.78	7.56	**6.51**

## 5 Conclusions

In this paper, a non-rigid face alignment method is proposed based on a single mismatched template. Considering two aspects about the matching rate and the matching number, a local matching method K-PP is presented based on mutual nearest neighbors searching. Furthermore, we employ the local matching features as a constraint for warping deformation parameters. The model fitting is demonstrated to be solved efficiently under the inverse compositional (IC) framework. Comparing to the conventional alignment method, the proposed method outperforms on the single template alignment.

Our contribution includes two main aspects: 1) A new local matching method K-PP is proposed which is robust to the complex facial deformation. 2) A formulation method for face alignment is proposed combining local and holistic matching. Both the flexible ability and robust ability are included which is useful for the non-rigid alignment problem when only given a single template. In the future work, more local features such as HOG features or Gaussian Filter will be used for illumination changing.
